# Peritumoral lymphoid cuff correlates well with lymph node enlargement in gastrointestinal schwannomas

**DOI:** 10.18632/oncotarget.24476

**Published:** 2018-02-09

**Authors:** Hyunsik Bae, Michael Van Vrancken, Tae Wook Kang, Ha Young Park, Jinah Chu, Hyung Kyu Park, Sang Yun Ha, Dongil Choi, Kyoung-Mee Kim

**Affiliations:** ^1^ Department of Pathology and Translational Genomics, Samsung Medical Center, Sungkyunkwan University School of Medicine, Seoul, Korea; ^2^ Department of Pathology, Christian Hospital, St. Louis, Missouri, USA; ^3^ Department of Radiology, Samsung Medical Center, Sungkyunkwan University School of Medicine, Seoul, Korea; ^4^ Department of Pathology, Inje University Busan Paik Hospital, Inje University School of Medicine, Busan, Korea

**Keywords:** schwannoma, gastrointestinal tract, lymphoid cuff, lymphadenopathy, subepithelial tumor, Pathology

## Abstract

**Background/Aims:**

To determine the incidence of regional lymphadenopathy in gastrointestinal (GI) schwannoma and to evaluate the relationship between peritumoral lymphoid cuff and lymphadenopathy.

**Methods:**

We queried 118 GI tract schwannomas and reviewed radiologic findings, intraoperative findings, and electronic medical records of all cases for enlarged regional lymph nodes.

**Results:**

Location of tumors included 85 gastric (72%), 11 colonic (9.3%), 7 esophageal (5.9%), 3 pancreatic (2.5%), 1 hepatic (0.8%), and 11 mesenteric (9.3%). The size of the tumors ranged from 0.2 to 11 cm (mean 3.8 cm). Histologically, 70.3% showed a peritumoral lymphoid cuff ranging in thickness from 0.3 to 6 mm (mean 1.6 mm). The peritumoral lymphoid cuff was significantly more frequent in gastric schwannomas (78.8%) followed by colonic (72.7%), esophageal (57.1%) and rare in other locations (*p* = 0.001). Of the 106 cases for which clinical or radiologic data was available for, 76 cases (71.7%) showed regional lymphadenopathy. The presence of peritumoral lymphoid cuff showed significant correlation with regional lymphadenopathy (*p* < 0.001) and the size of enlarged lymph nodes (*p* = 0.002).

**Conclusions:**

A peritumoral lymphoid cuff is frequently seen in GI tract schwannomas and correlates well with regional lymphadenopathy. However, in a significant subset (29.7%), a lymphoid cuff was not present warranting continued need for caution in the preoperative radiologic and postoperative pathologic diagnoses.

## INTRODUCTION

The differential diagnosis for subepithelial tumors of the gastrointestinal (GI) tract is broad and includes a wide range of benign and malignant entities including gastrointestinal stromal tumor (GIST), schwannoma, lymphoma, neuroendocrine tumor, leiomyoma, and metastatic carcinoma [1, 2]. Of these, GIST, schwannoma, and leiomyoma typically show spindle cell morphology. Among them, GIST is the most common, and the majority of them occur in the stomach [3]. Primary schwannomas of the GI tract are fairly uncommon accounting for only 0.2% of gastric tumors. However, similar to GISTs, they also share a predilection for the stomach making it one of the main differential diagnoses with GIST. A helpful histologic clue to the diagnosis of schwannoma is a peritumoral lymphoid cuff, which is rare in GIST [4].

In addition to their subepithelial location and similar histologic findings, schwannomas and GISTs also show similar macroscopic features [5]. Additionally, there are no distinct clinical characteristics that help separate the two which can lead to a misdiagnosis during preoperative evaluation [6–8]. However, their prognoses are markedly different making the distinction crucial [5, 9]. GI schwannomas usually follow a benign course and have an excellent prognosis following resection [4, 10], although rare cases of malignant transformation have been reported [11]. In contast, ~30% of GISTs have malignant behavior making local recurrence or metastases much more likely [12]. Therefore, the importance of an accurate preoperative diagnosis is crucial for the guidance of subsequent operative therapy. One report suggests that regional lymphadenopathy (LAD) is more commonly seen in schwannomas than GISTs. However, the underlying mechanism for such nodal enlargement has not been studied [13].

In this large series of GI tract schwannomas, we reviewed the clinical, pathologic, and radiologic findings of schwannomas and positively correlated the presence of a peritumoral lymphoid cuff with regional LAD.

## RESULTS

### Clinical findings

**Figure 1:**
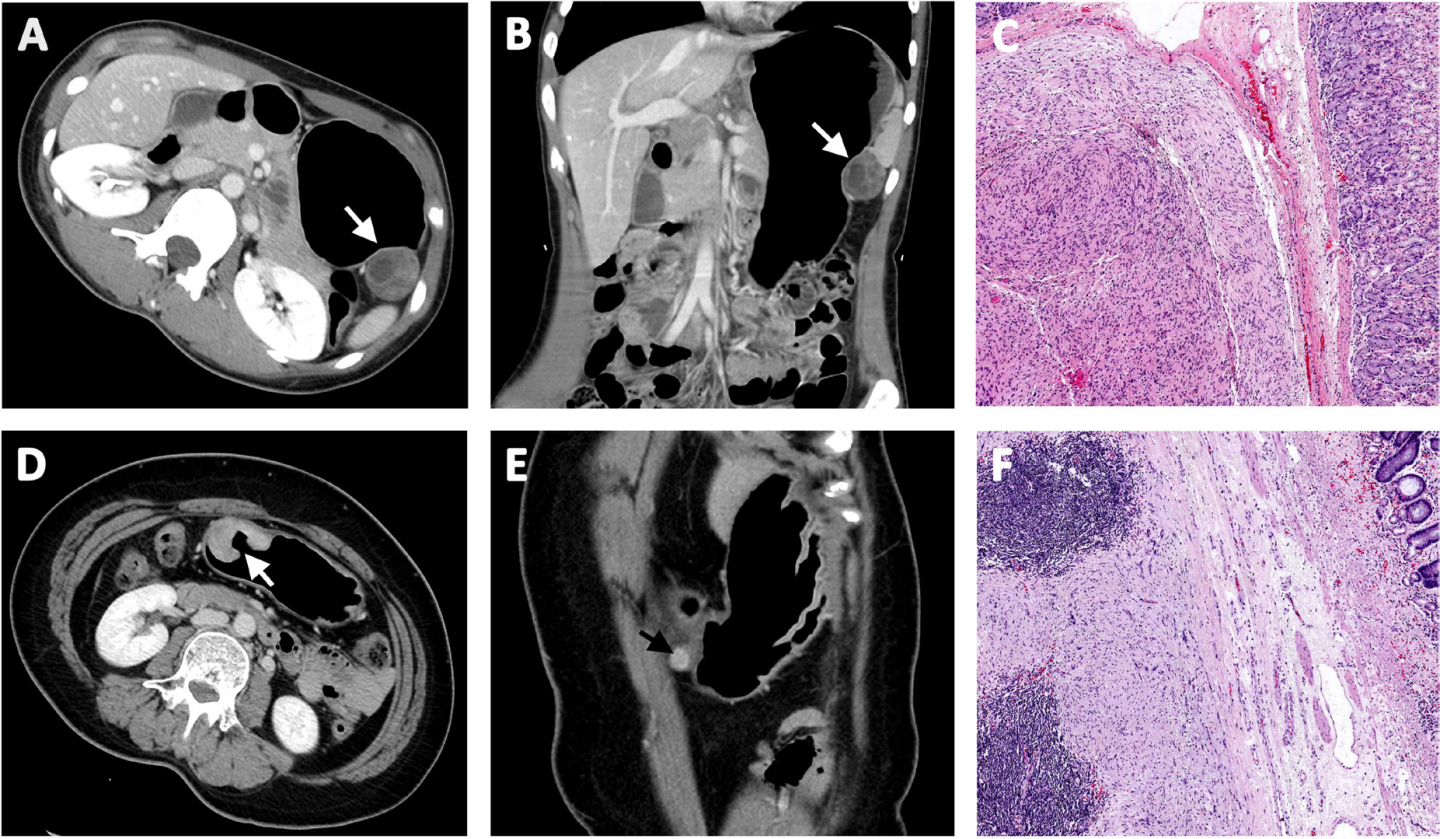
Representative preoperative computed tomographic image of gastric schwannoma (**A**) without regional lymphadenopathy (**B**) and corresponding pathologic findings without lymphoid cuff (**C**). A case of gastric schwannoma (**D**) with regional lymphadenopathy (**E**) and corresponding pathologic findings with lymphoid cuff (**F**).

Patients’ ages at diagnosis ranged from 25 to 80 years (mean, 55.5). The female to male ratio was 1.7:1. The anatomic location of GI tract schwannoma varied with gastric being the most common (85 cases; 72%) followed by colonic (11 cases; 9.3%), esophageal (7 cases; 5.9%), pancreatic (3 cases; 2.5%), hepatic (1 case; 0.8%), and intra-abdominal soft tissue around the GI tract (11 cases; 9.3%) (Table 1). 12 cases were incidentally found during other GI tract cancer surgery. 53 cases (44.9%) also underwent lymph node dissection due to preoperative radiologic findings of regional LAD. The representative preoperative computed tomographic images of gastric schwannoma with and without regional lymphadenopathy are depicted in Figure 1. A family history of neurofibromatosis type 1 or type 2 was not identified in any of the patients.

**Table 1:** Clinical, histological, and radiological characteristics of GI tract schwannoma

Sex			
	Male	44	(37.3%)
	Female	74	(62.7%)
**Location**			
	Stomach	85	(72.0%)
	Colon	11	(9.3%)
	Esophagus	7	(5.9%)
	Pancreas	3	(2.5%)
	Liver	1	(0.8%)
	Peritoneal soft tissue	11	(9.3%)
**Peripheral lymphoid cuff**			
	Present	83	(70.3%)
	Absent	35	(29.7%)
**Regional lymphadenopathy**			
	Present	76	(71.7%)
	Absent	30	(28.3%)

### Histopathologic findings

**Figure 2:**
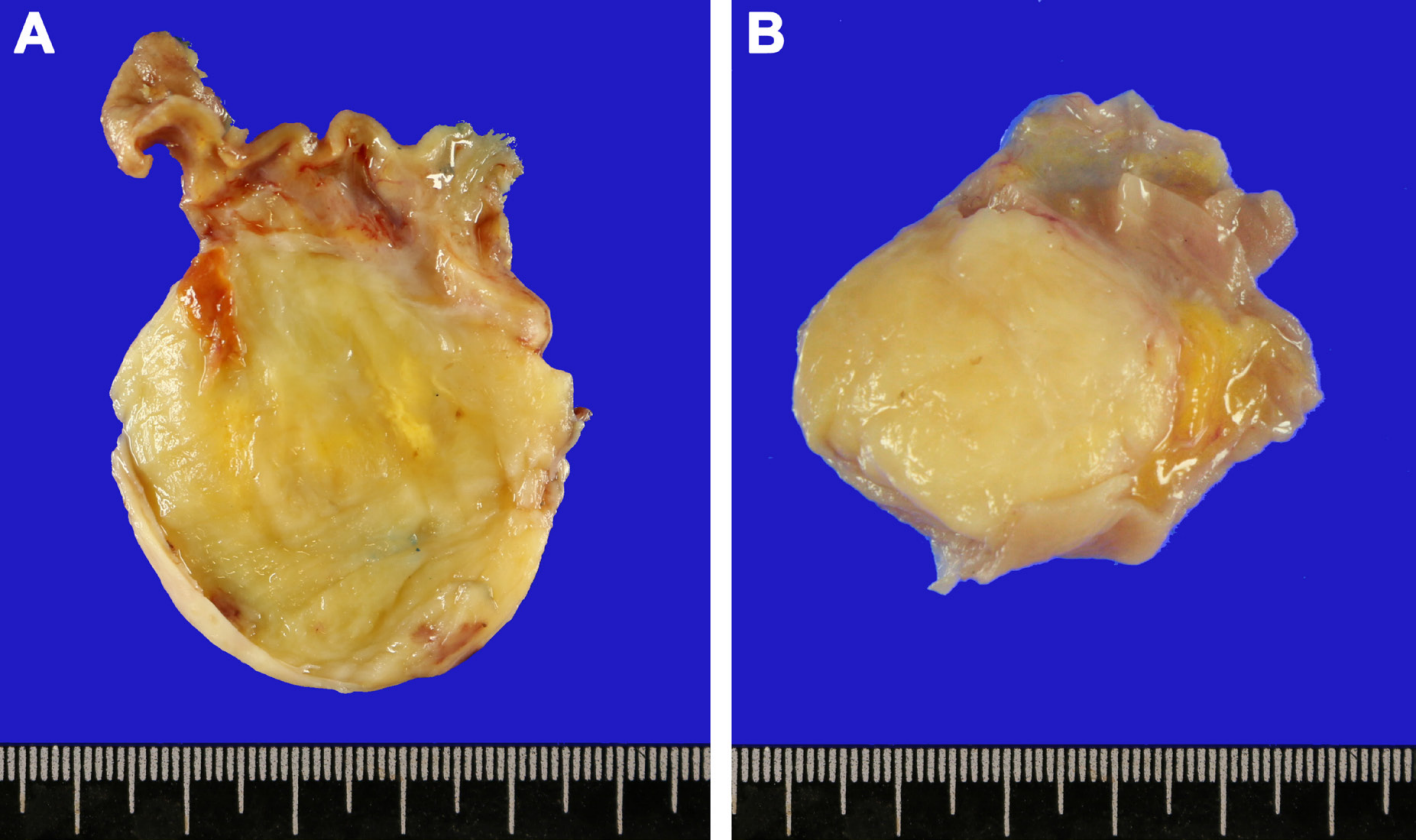
Gross images of gastrointestinal tract schwannomas located in the gastric lower body (**A**) and cecum (**B**). The cut section shows a well-circumscribed light yellow solid mass.

**Figure 3:**
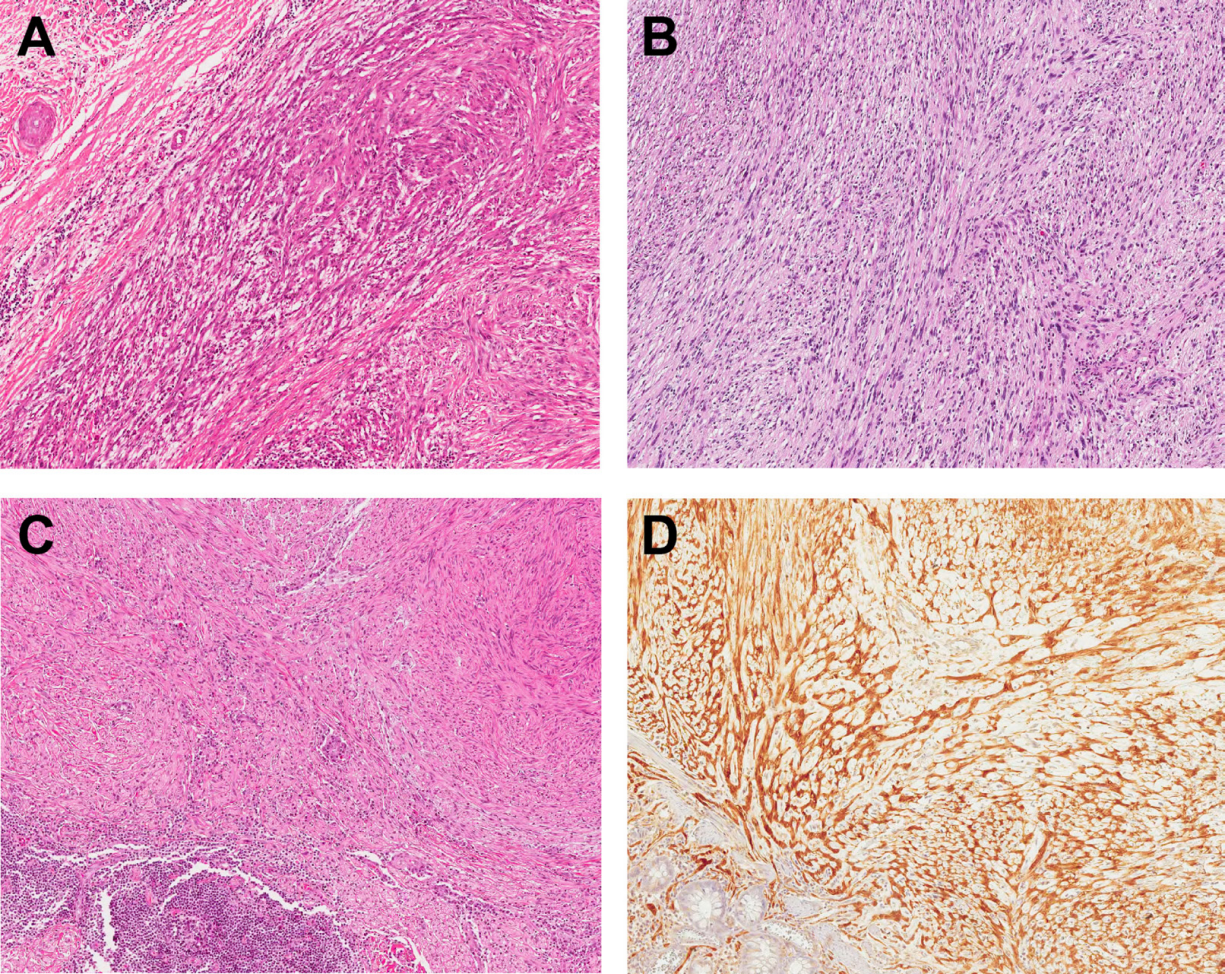
Histologic features of gastrointestinal tract schwannomas Tumor cells have frequent variation in cell size with spindle wavy nuclei and interspersed collagen fibers. Locations of schwannomas included stomach (**A**), rectum (**B**), and esophagus (**C**). Tumor cells are strongly positive for S-100 protein (**D**).

The size of the tumor ranged from 0.2 to 11 cm in greatest dimension with a mean of 3.8 cm. Cut section of the mass showed a tan to light yellow solid mass with occasional focal hemorrhage (Figure 2). Microscopically, the tumor cells had spindle wavy nuclei with tapered ends interspersed with collagen fibers (Figure 3). None of the GI schwannomas showed necrosis, atypical mitoses or invasion to mucosa and other adjacent organs. There was no evidence of local recurrence or metastasis during follow up (range 19–125 months; median 72 months). Most tumors were well demarcated (96%) with a nodular pattern (98%).

**Figure 4:**
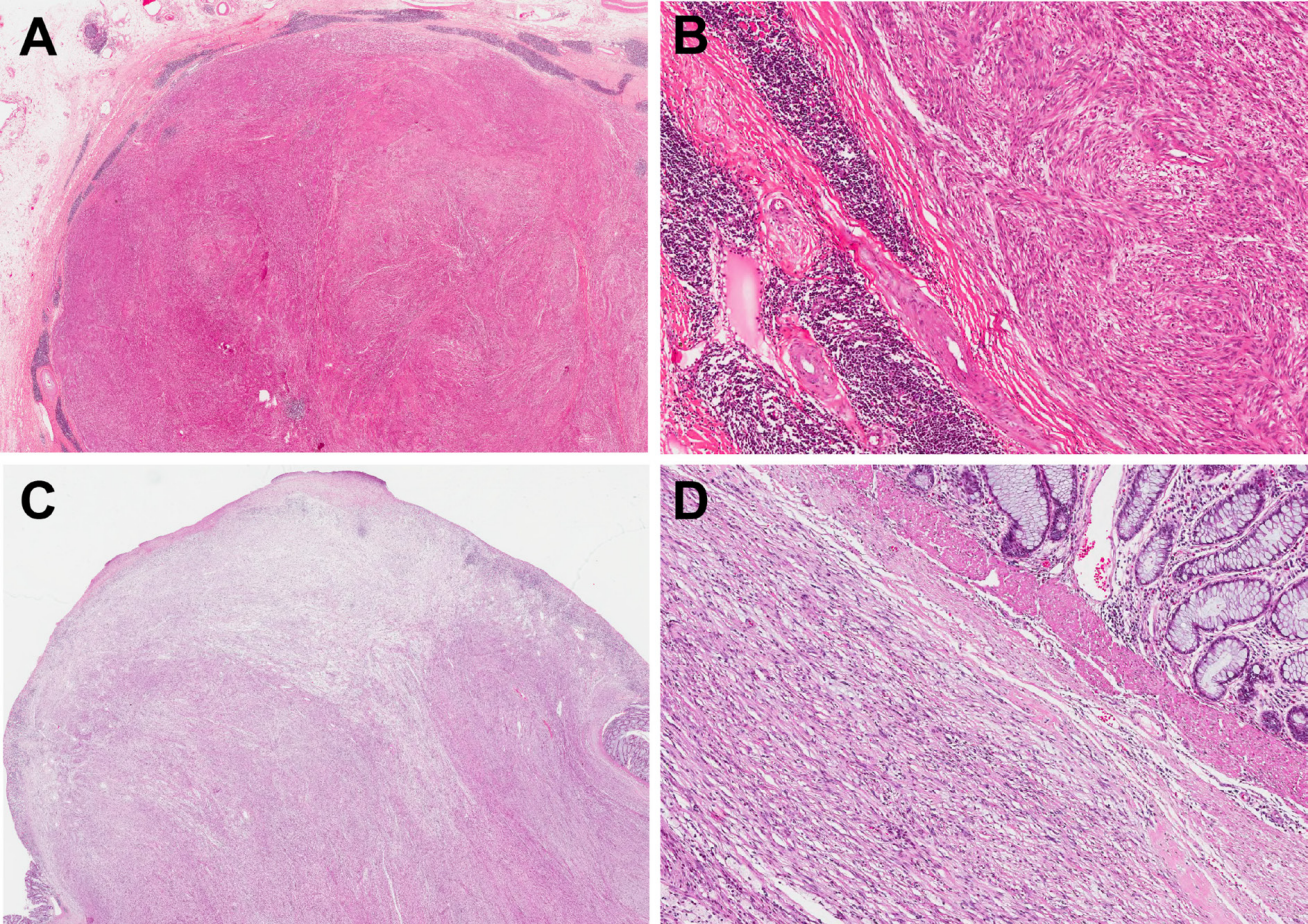
Peritumoral lymphoid cuff in schwannoma in the stomach (**A**, **B**), and colonic schwannoma without peritumoral lymphoid cuff (**C**, **D**).

**Figure 5:**
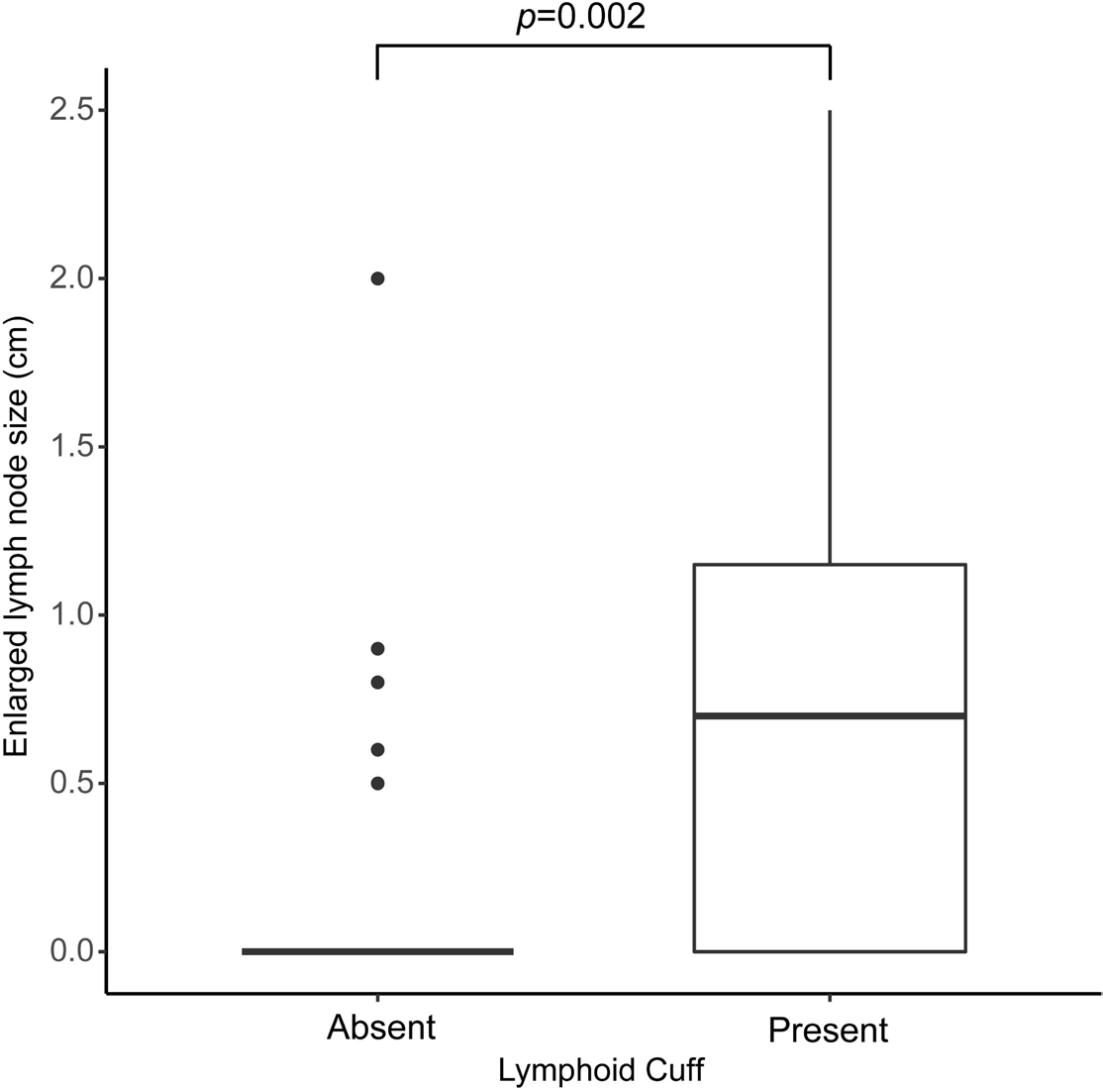
Size differences of regional lymphadenopathy in schwannomas with peritumoral lymphoid cuff and those without peritumoral lymphoid cuff (*p* = 0.002)

Histologically, 83 cases (70.3%) showed a peripheral peritumoral lymphoid cuff ranging in thickness from 0.2 to 6 mm (mean 1.63 mm) (Figure 4). Interestingly, the peritumoral lymphoid cuff was seen most frequently in gastric schwannomas (78.8%) followed by colonic (72.7%) and esophageal (57.1%) and rare in ones located in the liver and mesentery (33.3%). It was not present in pancreatic schwannomas (*p* = 0.001) (Table 2). Regional LAD was assessable through review of prior radiologic and concurrent pathologic findings after operation in all but 12 cases. Out of the 106 available cases, 76 cases (71.7%) showed regional LAD. Regional lymph node dissection was performed in 41 cases (38.7%). The pathology of resected enlarged lymph nodes showed sinus hyperplasia (22 cases; 53.7%) and lymphoid follicular hyperplasia (19 cases; 46.3%). The presence of the peritumoral lymphoid cuff and regional LAD showed a statistically significant positive correlation (*p* < 0.001) (Table 3). Moreover, there was a significant difference in the size of enlarged lymph nodes between tumors with a peritumoral lymphoid cuff (mean size 7.9 mm) and without a lymphoid cuff (mean size 2.3 mm) (*p* = 0.002) (Figure 5).

**Table 2:** Correlation of anatomic site of tumor and peripheral lymphoid cuff

Anatomic site	Peripheral lymphoid cuff		
	Absent	Present	
**Stomach**	18 (21.2%)	67 (78.8%)	*p* = 0.001
**Colon**	3 (27.3%)	8 (72.7%)	*p* > 0.05
**Esophagus**	3 (42.9%)	4 (57.1%)	*p* > 0.05
**Pancreas**	3 (100%)	0 (0%)	*p* > 0.05
**Liver and Peritoneal soft tissue**	8 (66.7%)	4 (33.3%)	*p* > 0.05

**Table 3:** Correlation of lymphoid cuff and regional lymph node enlargement

		Lymph node enlargement		
		No	Yes	
**Lymphoid cuff**	**Absent**	19	7	
		(73.1%)	(26.9%)	
	**Present**	11	69	
		(13.8%)	(86.2%)	*p* < .001

## DISCUSSION

In this first and largest series of GI tract schwannomas, we found that a peritumoral lymphoid cuff is frequently seen in GI tract schwannomas, and it correlates highly with the presence and size of regional LAD. However, it should be noted that in a significant subset (29.7%) a lymphoid cuff was not present in schwannomas of the GI tract.

The differential diagnosis of subepithelial tumors of the GI tract includes GIST, leiomyoma, lymphoma, and schwannoma. Among these lesions, GIST is the most common tumor with the majority of them occurring in the stomach. Primary schwannomas of the GI tract are a rare subepithelial tumor also occurring most often in the stomach thus making it an important differential diagnosis for GISTs. They account for 0.2% of all gastric tumors and 4% of all benign gastric neoplasms [5]. As both GIST and schwannoma originate from mesenchyme and have a similar morphology, their distinction can be difficult. As further evidence of this, 5 GI tract schwannoma cases were actually misdiagnosed as GISTs due to their histologic similarities at our institute. One frequent and typical histologic finding seen in GI schwannomas is a peripheral peritumoral lymphoid cuff which is not typically associated with GIST. Other distinguishing features of GI schwannoma include frequent tumor cell size variation, absence of skeinoid fibers, S-100 protein positivity (100% versus <20% in GIST), and c-kit (CD117) negativity [9]. Additionally, as DOG1 is a relatively specific immunohistochemical for GIST, in equivocal cases of GI schwannomas without a peritumoral lymphoid cuff, a DOG1 stain was also performed to entirely exclude a mimicking GIST. All schwannomas with no peritumoral lymphoid cuff were DOG1 negative.

Due to the rarity, there are relatively few case studies and literature reviews of primary GI tract schwannomas [14–21]. Most of these previous studies focused on the rarity of tumor, immunohistochemical findings, and differential diagnostic points from other mesenchymal tumors. Tao, *et al.*, reported 30 cases of primary gastric schwannoma seen at a single institute over 8 years and found a peritumoral lymphoid cuff in 26 patients (86.7%) [22]. This incidence is similar to our observations (78.8%). In this study, we collected 118 cases of GI tract schwannomas including 85 gastric during an 18 year period and analyzed periturmoral lymphoid cuff according to anatomic location and its relationship with regional LAD.

In 2002, our institute reported 12 cases of GI schwannomas collected from 1995 to 2001 with regards to clinicopathological features and to the differential diagnosis with GIST [9]. In 2012, we reported a series of small (5 cm or smaller) gastric submucosal tumors collected from 2004 to 2008. We found 16 schwannomas out of 96 (16.7%) tumors and reported them with special emphasis on the differential diagnosis with GISTs and imaging findings (CT scan) [13]. In the present study, we collected all of these prior GI schwannoma cases and observed the presence and thickness of a peritumoral lymphoid cuff and correlated this with adjacent lymphadenopathy. In preoperative radiologic studies, regional LAD has proved to be a somewhat useful finding helping to distinguish between GI schwannomas from other types of subepithelial tumors (particularly GISTs) [23, 24]. However, most prior studies were focused on gastric cases, and the underlying mechanism responsible for the LAD or other possible associations with the LAD has not been adequately studied. In this study, we observed that peritumoral lymphoid cuff is positively and significantly correlated with the presence and size of regional LAD. Peritumoral lymphoid cuffs were seen in 70.3% of GI tract schwannomas. It is noteworthy that almost half of the cases underwent lymph node dissection due to preoperative radiologic findings of regional LAD in patients with GI schwannoma. Interestingly, in a significant subset (29.7%) lymphoid cuffs were not present warranting continued need for caution in the preoperative radiologic and pathologic diagnoses of schwannoma.

In conclusion, a peritumoral lymphoid cuff is frequently seen in GI tract schwannomas, however, in a significant subset, a lymphoid cuff was not present warranting continued need for caution in the preoperative radiologic and postoperative pathologic diagnoses.

## MATERIALS AND METHODS

The Samsung Medical Center pathology database was queried for all resected gastrointestinal schwannomas from January 1998 to May 2016. Two pathologists (H.B and K.-M.K.) reviewed all cases. The pathologic diagnosis was defined as a well circumscribed lesion with an admixture of compact cellular spindled (Antoni A) and hypocellular (Antoni B) areas with collagen fiber deposition and positive expression for S-100 protein (polyclonal antiserum, 1:40 dilution, Zymed, San Francisco, CA, USA) and negative for c-kit (polyclonal antiserum, 1:40 dilution, Santa Cruz Biotechnology, Santa Cruz, CA, USA) by immunohistochemistry. In cases suspicious for schwannoma but lacking a peritumoral lymphoid component, we also applied DOG1 (clone K9, Leica Microsystems, Wetzlar, Germany, 1:200) by immunohistochemistry to completely exclude GIST. Finally, a total of 118 GI schwannoma cases were identified. Among them, 5 cases were initially misdiagnosed as c-kit negative GIST because of their histologic similarity and absence of lymphoid cuff within the tumor. Written informed consent was obtained from all patients.

All resected tissues were fixed overnight in 10% buffered formalin and were embedded in paraffin. Hematoxylin and eosin-stained tissue sections (4 μm) of all cases were examined, and the characteristics of anatomic location, tumor border, nodularity, peritumoral lymphoid cuff, and regional LAD status were collected. Tumor size, the thickest area of peritumoral lymphoid cuff, and the length of the short axis of enlarged lymph nodes were also measured. For correlation, experienced radiologists (T.W.K. and D.C.) reviewed all the computed tomographic (CT) images and reports. Enlarged lymph nodes were defined as >4 mm of its length of short axis when measured in CT images in the picture archiving and communication system (PACS) workstation (PathSpeed, GE Healthcare, Milwaukee, WI, USA). When two or more preoperative CT scans were performed, the most recent scan was used for analysis. Also we reviewed intraoperative findings, and electronic medical records of all cases describing the presence of enlarged regional lymph nodes.

Statistical analysis was performed using SPSS version 23.0 software (IBM, NY, USA). Significant differences were evaluated using Fisher's exact test for categorical data. A *p* < 0.05 was considered to indicate a statistically significant difference.
